# Relationship Between Cerebral Oxygenation and Metabolism During Rewarming in Newborn Infants After Therapeutic Hypothermia Following Hypoxic-Ischemic Brain Injury

**DOI:** 10.1007/978-3-319-38810-6_33

**Published:** 2016-05-02

**Authors:** Subhabrata Mitra, Gemma Bale, Judith Meek, Cristina Uria-Avellanal, Nicola J. Robertson, Ilias Tachtsidis

**Affiliations:** 10 Institute for Women’s Health, University College London and Neonatal Unit, University College London Hospitals Trust, London, UK; 11 Biomedical Optics Research Laboratory, Department of Medical Physics and Biomedical Engineering, University College London, London, UK

**Keywords:** Hypoxic-ischaemic brain injury, Cerebral oxygenation, Cerebral metabolism, Cytochrome-c-oxidase, Newborn infant

## Abstract

Therapeutic hypothermia (TH) has become a standard of care following hypoxic ischemic encephalopathy (HIE). After TH, body temperature is brought back to 37 °C over 14 h. Lactate/N-acetylasperatate (Lac/NAA) peak area ratio on proton magnetic resonance spectroscopy (^1^H MRS) is the best available outcome biomarker following HIE. We hypothesized that broadband near infrared spectroscopy (NIRS) measured changes in the oxidation state of cytochrome-c-oxidase concentration (Δ[oxCCO]) and cerebral hemodynamics during rewarming would relate to Lac/NAA. Broadband NIRS and systemic data were collected during rewarming from 14 infants following HIE over a mean period of 12.5 h. ^1^H MRS was performed on day 5–9. Heart rate increased by 20/min during rewarming while blood pressure and peripheral oxygen saturation (SpO_2_) remained stable. The relationship between mitochondrial metabolism and oxygenation (measured as Δ[oxCCO] and Δ[HbD], respectively) was calculated by linear regression analysis. This was reviewed in three groups: Lac/NAA values <0.5, 0.5–1, >1. Mean regression coefficient (*r* ^2^) values in these groups were 0.41 (±0.27), 0.22 (±0.21) and 0.01, respectively. The relationship between mitochondrial metabolism and oxygenation became impaired with rising Lac/NAA. Cardiovascular parameters remained stable during rewarming.

## Introduction

Perinatal hypoxic-ischaemic (Hypoxic-ischaemic (HI))TH Therapeutic hypothermia (TH) brain injury causes significant morbidity and mortality. Therapeutic hypothermia (TH) is beneficial following hypoxic ischemic encephalopathy (HIE) and has become the standard of care in recent years [1, 2]. During TH, body temperature is maintained at 33.5 °C followed by a slow rewarming that brings body temperature back to 37 °C. Rewarming early from TH induces cortical neuron apoptosis in a piglet model following HIE [3]. Rebound seizures have been noted during the rewarming period both in animal models [4] and neonatal intensive care [5] and further ‘cooling’ with slower rewarming has been suggested. Changes in Therapeutic hypothermia (TH) and Therapeutic hypothermia (TH) have been investigated in both preclinical models and clinical studies during and after Hypoxic-ischaemic (HI), but the dynamic effects of rewarming on newborn cerebral metabolism and hemodynamics have not yet been fully investigated.

NIRS is a non-invasive tool that has been widely used for continuous bedside monitoring of cerebral oxygenation and hemodynamic changes. We have recently developed a new broadband NIRS system to monitor Δ[oxCCO] as well as the concentration changes of oxy- and deoxy hemoglobin (Δ[HbO_2_] and Δ[HHb], respectively) in neonatal brain [6]. Cytochrome-c-oxidase (CCO) plays a crucial role in mitochondrial oxidative metabolism and ATP synthesis and is responsible for more than 95 % of oxygen metabolism in the body [7]. Changes in total hemoglobin (HbT = HbO_2_ + HHb) and hemoglobin difference (HbD = HbO_2_ − HHb) were calculated. Changes in HbD and HbT reflect changes in cerebral oxygenation and changes in cerebral blood volume, respectively.

Following HIE, Lac/Lactate/N-acetylasperatate (Lac/NAA) peak area ratio obtained from Therapeutic hypothermia (TH) is the best available MR biomarker for prediction of neurodevelopmental outcome [8].

The aim of this study was to assess the cerebral metabolic and hemodynamic changes during the rewarming period in a cohort of term infants following perinatal hypoxic-ischemic brain injury. We hypothesized that the dynamic changes in cerebral oxygenation and metabolism during the rewarming period would relate to the severity of the injury as assessed by Lac/NAA.

## Methods

Ethical approval for the study at University College London Hospitals NHS Foundation Trust (UCLH) was obtained from the NREC (reference: 13/LO/0225). Hypothermia and subsequent rewarming were instituted with a servo-controlled cooling machine Tecotherm Neo, Inspiration Healthcare, UK. Using a cooling mattress and a rectal temperature probe, it maintains a constant core temperature during hypothermia and increases the temperature as programmed during the rewarming period. Normally, the core temperature is increased by 0.5 °C over every 2-h period so that the temperature is increased from 33.5 to 37 °C over 14 h. Rewarming data were collected from a cohort of 14 infants. NIRS monitoring was commenced as early as possible in the rewarming phase and was continued for the maximum possible time. One NIRS channel was placed on either side of the forehead and data were collected at 1 Hz. The Therapeutic hypothermia (TH) distance of 2.5 cm was chosen to ensure a good depth penetration [9]. The differential path length (DPF) was chosen as 4.99 [10]. Changes in chromophore concentrations were calculated from the measured changes in broadband NIR light attenuation using the modified Beer-Lambert law as applied with the Therapeutic hypothermia (TH) [11] across 136 wavelengths (770–906 nm). Systemic data were collected using ixTrend software (ixellence GmbH, Germany) and were synchronised with the NIRS data. Brain magnetic resonance imaging (MRI) and MRS were performed between day 5–9 using a 3T Philips MRI scanner (Philips Healthcare, UK).

## Data Analysis

Initial data analysis was carried out in MATLAB (Mathworks, USA). Systemic data were down-sampled and interpolated to the NIRS data timeframe (1 Hz). Artefacts from movement or changes in external lighting were removed and baseline shifts were corrected using the method suggested by Scholkmann et al. [12]. Linear regression analysis was performed to assess the relationship between Δ[oxCCO] and Δ[HbD] and an averaged regression coefficient (*r* ^2^) was created to compare this relationship between groups. All statistical analyses were performed using GraphPad Prism 6 (GraphPad Software, USA).

## Results

**Fig. 33.1:**
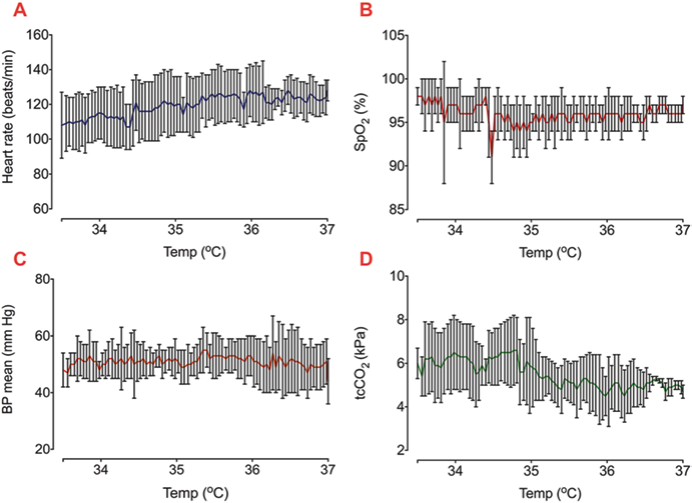
Systemic changes (heart rate (**a**), SpO_2_ (**b**), mean blood pressure (**c**) and transcutaneous CO_2_ (**d**)) among all infants during Therapeutic hypothermia (TH) (mean ± s.d.)

**Fig. 33.2:**
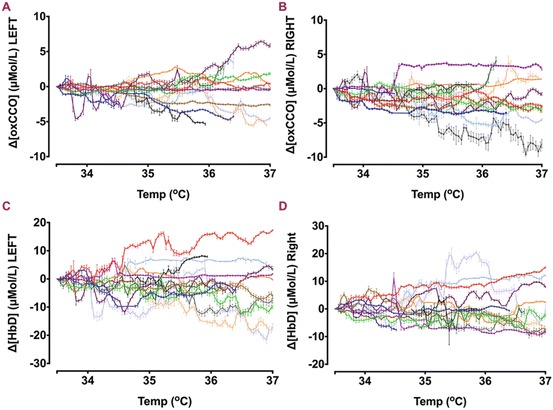
Δ[oxCCO] (**a**, **b**) and Δ[HbD] (**c**, **d**) from both left and right sides during Therapeutic hypothermia (TH). Each individual color represents data from one infant

**Fig. 33.3:**
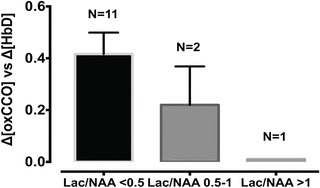
Averaged regression coefficient (*r* ^2^) between oxCCO and HbD presented in three groups: Lac/Lactate/N-acetylasperatate (Lac/NAA) <0.5, 0.5–1 and >1

NIRS and systemic data were collected over a mean period of 12.5 h (5–14 h). Active cooling was started at a mean of 3 h of age. All infants were ventilated during rewarming. Clinical characteristics of the cohort are presented in Table 33.1. During rewarming the heart rate gradually increased from a mean of 108/min (range 75–130/min) to 128/min (121–135/min). The peripheral oxygen saturation (SpO_2_) fell briefly at the start of Therapeutic hypothermia (TH), but was mostly over 95 %. Mean blood pressure (BP) was stable throughout the rewarming (45–55 mmHg) (Fig. 33.1). Transcutaneous CO_2_ dropped by mean 1.3 kPa. Changes in oxCCO concentration from both left and right side are shown in Fig. 33.2. No significant difference was noted between the two sides. The averaged regression coefficients (*r* ^2^) between Δ[oxCCO] and Δ[HbD] were plotted against Lac/Lactate/N-acetylasperatate (Lac/NAA) values obtained from ^1^H MRS. Although in clinical practice a Lac/NAA peak area ratio >0.3 has been associated with poor neurodevelopmental outcome following HIE [8], we divided the current cohort into three groups (Lac/NAA <0.5, 0.5–1 and >1) to more explicitly demonstrate the relationship between the average regression coefficient between Δ[oxCCO] and Δ[HbD] with Therapeutic hypothermia (TH). Lac/NAA peak area ratio ranged from 0.08 to 1.32 in this cohort, and four infants had a value of ≥0.3. Eleven infants had Lac/NAA <0.5, 2 had Lac/NAA 0.5–1 and 1 had Lac/NAA > 1. Mean *r* ^2^ values between Δ[oxCCO] and Δ[HbD] in these groups were 0.41 (±0.27), 0.22 (±0.21) and 0.01, respectively (Fig. 33.3).

**Table 33.1:** Characteristics of the infants: data are presented as mean (range)

Infant characteristics	Mean (range)
Birth weight (g)	3161 (1770–3800)
Gestational age (weeks)	39 (38–41)
Male:female	1.3:1
Apgar score at 1 min	1 (0–4)
Apgar score at 5 min	3 (0–7)
Apgar score at 10 min	3 (0–8)
Umbilical cord pH (arterial)	6.9 (6.56–7.28)
Base excess (arterial)	−15 (9.7–24)
Serum lactate	12 (9–17)
Age at start of active cooling (min)	178 (55–284)

## Discussion

The relationship between cerebral metabolism and oxygenation measured during rewarming following TH in a group of infants with HIE became more disturbed with an increasing degree of brain injury. Rewarming is a complex process; it has the potential to significantly influence cardiovascular function and stimulate the activity of excitatory amino acids suppressed during hypothermia [13].

Gebaur et al. reported that during hypothermia left ventricular output remains low due to decreased heart rate and a decreased stroke volume. During rewarming both stroke volume and cardiac output increases as the core temperature increases [14]. In our study, heart rate increased steadily throughout rewarming but mean BP remained stable, no hypotensive episodes were noted. Our findings were similar to those of Gebauer et al. but were different from those reported by Thoresen and Whitlaw [15]. They noted changes in cardiovascular indices in 9 infants with Therapeutic hypothermia (TH) during mild hypothermia and rewarming. Mean arterial BP fell during rewarming while the heart rate increased. This difference is probably related to the process of rewarming. In the study described by Thoresen et al. rewarming was performed over a minimum of 5 h by removing the cooling cap and adjusting the overhead heater to control the rise of temperature no more than per hour. We used a servo controlled cooling machine, which increased the core temperature in a more stable way, and the rewarming was done over a much longer period. A rapid and unstable increase in core temperature most likely induces a reduced peripheral vascular tone with increased cardiac work. Rewarming at a rate of 0.5–1 °C/h did not influence the beneficial effects of therapeutic hypothermia in a rat model, but a higher rewarming rate of 2 °C/h abolished those beneficial effects on both cardiac and cerebral function (reduced severity of myocardial and cerebral functions abnormalities and attenuated release of IL-6 and TNF-α) [16].

Availability of oxygen has a significant influence on the oxidation of CCO. The relationship between Therapeutic hypothermia (TH) became more impaired with increasing severity of injury, measured as rising Lac/Lactate/N-acetylasperatate (Lac/NAA) on ^1^H MRS. This probably indicates that following severe HIE and cell death, cerebral metabolism failed to improve in spite of oxygen availability. We did not notice any significant pattern of changes in Δ[oxCCO] with a change in temperature, nor did we notice any specific cut-off temperature point which indicated any change in the trend of cerebral metabolism during rewarming. In near-term fetal sheep, carotid artery blood flow (CaBF) and mean arterial blood pressure (MABP) changed only transiently during rewarming. No significant difference was noted from 6 h onwards [3]. In a recent study, asphyxiated newborn infants had stable regional cerebral oxygenation during rewarming [17].

The limitation of the present study is the small number of infants enrolled and, in particular, the number of infants with severe brain injury.

We noted that the mitochondrial metabolism-oxygenation coupling during rewarming was influenced by the severity of Therapeutic hypothermia (TH). Servo-controlled slow rewarming process had no significant influence on the stability of cerebrovascular hemodynamics and metabolism.
